# Use of single-item scales in the evaluation of German digital health applications (DiGAs): Scoping review protocol

**DOI:** 10.1371/journal.pone.0356485

**Published:** 2026-08-26

**Authors:** Julian Wienert, Mahin Mohammadi

**Affiliations:** 1 IU International University of Applied Sciences, Erfurt, Germany; 2 Independent Researcher, Iran; Gulu University, UGANDA

## Abstract

**Introduction:**

German Digital Health Applications (DiGAs) are reimbursable, patient-facing digital interventions within the German healthcare system that must demonstrate positive healthcare effects. Outcome measurement in DiGA evaluations is influenced by both methodological considerations and feasibility requirements in real-world care settings. In practice, these evaluations may be affected by factors such as limited patient adherence, high dropout rates, and variability in real-world data quality, which can complicate outcome data collection. Single-item scales may offer a low-burden alternative to multi-item instruments; however, their use, justification, and limitations in DiGA evaluations have not been comprehensively mapped. This paper presents a scoping review protocol designed to address this gap.

**Methods and analysis:**

This protocol outlines a scoping review that will follow the Joanna Briggs Institute methodology and be reported in accordance with PRISMA-ScR guidelines. The review will apply the PCC framework, focusing on DiGAs listed in the BfArM DiGA directory, the use of single-item scales, and evaluations conducted within the German healthcare system. Studies will be identified primarily through the DiGA directory and supplemented by PubMed searches to verify and complete bibliographic information. Two reviewers will independently screen and verify extracted data. Quantitative data will be summarized descriptively, while qualitative information on constructs, rationales, and methodological considerations will be analyzed thematically.

**Results:**

As this is a protocol, no results are reported. The planned review will provide a descriptive overview of single-item scale use in DiGA evaluations, map the constructs assessed, and identify methodological characteristics and gaps.

**Conclusion:**

This protocol outlines a transparent and reproducible approach to mapping outcome measurement practices in DiGAs. The findings of the completed review will inform researchers, developers, and policymakers and support methodological development in regulated digital health contexts.

**Registration:**

The scoping review protocol is registered at the Open Science Framework under the following DOI https://doi.org/10.17605/OSF.IO/A3PM5.

## Introduction

Digital health applications are increasingly integrated into routine healthcare delivery. In Germany, German Digital Health Applications (Digitale Gesundheitsanwendungen; DiGAs) represent a distinct category of patient-facing digital health applications. These applications can be prescribed by medical doctors and are reimbursed by statutory health insurance schemes once they are listed in the official DiGA directory maintained by the German Federal Institute for Drugs and Medical Devices (BfArM) [1]. The DiGA directory is a regulatory register of all Digital Health Applications that have successfully completed the statutory assessment procedure under §139e SGB V and the German Digital Health Applications Ordinance (DiGAV). It provides structured information on each DiGA, including basic product characteristics (e.g., name, manufacturer, intended medical purpose), functional description, usage conditions, data protection and interoperability features, and information relevant to healthcare providers and patients. Importantly, it also contains references to the scientific evidence submitted for reimbursement approval, including evaluation studies and trial registrations used to demonstrate positive healthcare effects.

As part of the DiGA fast-track process, applications must meet requirements related to safety, functionality, data protection, and interoperability. They must also demonstrate a positive healthcare effect to achieve and maintain reimbursement [1,2]. Positive healthcare effects may consist of either medical benefits or patient-relevant improvements in care processes, such as improved health management, health-related quality of life, or patient empowerment [3]. Consequently, the evaluation of DiGAs frequently employs randomized controlled study designs that are implemented in field-based or routine care settings rather than in tightly controlled experimental environments [3]. Measuring outcomes in digital care requires balancing methodological rigor with practical feasibility to ensure data quality [3,4].

In this context, single-item scales offer a promising approach to mitigating competing demands between methodological rigor and feasibility. A single-item scale is a measurement instrument consisting of only one question or statement to assess a particular construct or attribute. Despite their simplicity, these scales can be effective for measuring various aspects, such as symptoms, satisfaction, or perceived health status, particularly in contexts where brevity is essential, such as in digital health intervention [5–7]. For example, a commonly used single-item measure is the global health question: “In general, would you say your health is excellent, very good, good, fair, or poor?” [5]. Recent evidence from digital mental health contexts further supports the practical utility of single-item scales for large-scale screening, outcome monitoring, and integration into digital platforms. This review provides a valuable synthesis of single-item scales use in digital mental healthcare from a clinical effectiveness perspective [8]. More recent clinical research further supports the utility of single-item quality-of-life assessments, demonstrating robust associations with well-being, health indicators, and lifestyle-related correlates [6]. Methodological studies further suggest that single-item measures may be appropriate when constructs are unidimensional, concrete, and easily understood by respondents, particularly when minimizing participant burden is a priority [7].

At the same time, single-item scales entail important methodological limitations. Due to their limited item content, they may not adequately capture complex or multidimensional latent constructs. They do not allow for the assessment of internal consistency reliability and may be more sensitive to wording and contextual interpretation effects. Their validity and interpretability may therefore vary across populations, settings, and constructs, which can limit their suitability for detailed outcome evaluation [9,10]. Consequently, their use often requires careful theoretical justification and transparent reporting, particularly in contexts where measurement quality informs regulatory or reimbursement-related decision-making [11].

Despite evidence on both advantages and limitations in health-related research, the use of single-item scales in DiGA evaluations has not been systematically mapped. It remains unclear which constructs are assessed using single-item measures and how their use is justified within this regulated reimbursement context. This scoping review addresses this gap by systematically mapping single-item scale use across DiGA evaluation studies.

### Aims and objectives

The purpose of this scoping review is to systematically identify and characterize the use of single-item scales in evaluation studies of DiGAs.

### Objectives

This review will address the following objectives:

- To identify which DiGA evaluations employ single-item scales.
- To describe the constructs assessed using single-item scales.
- To map the reported rationales, advantages, and limitations associated with their use.
- To identify methodological patterns and gaps in current evaluation practices.

### Expected outcomes

The findings of this review are expected to provide (1) a descriptive overview of single-item scale use across DiGAs, (2) an exploratory mapping of the constructs assessed, and (3) a preliminary identification of methodological patterns and potential gaps in current practices. These outcomes are intended to inform researchers, developers, and policymakers and to support future methodological development in regulated digital health applications.

## Methods and analysis

The protocol for this scoping review has been submitted to the Open Science Framework (OSF) platform, where it has been registered and received the following DOI https://doi.org/10.17605/OSF.IO/A3PM5.

The scoping review will be conducted in accordance with the criteria specified in this protocol, alongside the PRISMA (Preferred Reporting Items for Systematic Reviews and Meta-Analyses) guidelines for scoping reviews, particularly the PRISMA-ScR extension and its associated flow diagram [12]. Furthermore, the study methods were developed in line with OSF guidelines [13,14]. The overall approach is grounded in Arksey and O’Malley’s scoping review framework, incorporating subsequent methodological refinements proposed by Levac et al. as suggested by the Joanna Briggs Institute (JBI) methodology for scoping reviews [15–17].

The framework comprises six key stages:

i Identifying the research question
ii Identifying relevant studies
iii Study selection
iv Charting the data
v Collating, summarizing and reporting the results
vi Consultation

A flow diagram will also be produced for the scoping review to illustrate the progression of information throughout the various stages of the review. This will detail the number of records identified, screened, included, and excluded, along with the reasons for exclusion.

### Identifying the research question

The research question guiding this scoping review is: How are single-item scales used in the evaluation of DiGAs, and what constructs, rationales, advantages, and limitations are reported in the literature? This question was developed using the Population–Concept–Context (PCC) framework [13], which serves as the foundation for our eligibility criteria:

#### Population.

DiGAs, defined as patient-facing digital health interventions listed in the BfArM DiGA directory.

#### Concept.

The use of single-item scales in the evaluation of DiGAs, including their purpose, the constructs assessed, and reported advantages or limitations.

#### Context.

The evaluation of DiGAs conducted within the German healthcare system, including studies performed as part of the DiGA fast-track process or for reimbursement-related evidence generation.

Clinical indications of DiGAs (e.g., psychiatry, cardiology, dermatology) are not used as analytical strata in this review, as the focus is exclusively on measurement methodology, specifically the use of single-item scales, across DiGAs as a unified regulatory sampling frame within the German reimbursement system, while the use of single-item measures constitutes the primary unit of analysis and the central focus of this scoping review.

### Identifying relevant studies

The primary information source for this scoping review will be the official DiGA directory maintained by the BfArM, which provides a comprehensive and authoritative listing of all reimbursable Digital Health Applications in Germany. As this directory constitutes the complete regulatory register of DiGAs, all DiGAs listed in the directory will be included.

For each DiGA, associated evaluation studies and/or trial registrations required for reimbursement approval are already listed within the DiGA directory and will serve as the primary source for identifying relevant evidence. Bibliographic details, study identifiers, and available publication information will be extracted directly from these entries.

A supplementary search in PubMed will be conducted only where bibliographic information is incomplete, unclear, or outdated, or where study entries do not provide sufficient detail to identify the corresponding publication. In such cases, PubMed will be used to verify and complete publication metadata associated with the listed evaluation studies.

Boolean search operators will only be applied in PubMed when necessary to retrieve missing or ambiguous records, using DiGA names and/or trial registration identifiers as search terms. Authors may be contacted if required to resolve remaining uncertainties. All searches will include studies available up to the date of extraction. The retrieved evaluation studies will subsequently be assessed for eligibility.

All procedures for extraction, verification, and supplementation of bibliographic information will be systematically documented to ensure transparency, reproducibility, and auditability. This registry-based approach ensures comprehensive coverage of all reimbursable DiGAs and their associated evaluation studies while minimizing unnecessary database searching and maintaining high data integrity. Finally, all records will be transferred to the CADIMA platform, which will be used to manage study selection, screening, and documentation of the review process.

### Selecting eligible studies (inclusion and exclusion criteria)

This scoping review will utilize the PCC framework to establish inclusion and exclusion criteria [13]. Screening will be conducted in two stages by human reviewers. First, all DiGAs and linked studies from the BfArM DiGA directory will be screened for relevance based on titles and available bibliographic information. In a second stage, full texts or abstracts retrieved from PubMed will be assessed against inclusion and exclusion criteria, with any disagreements resolved through discussion. As studies will be extracted from the BfArM DiGA directory, no duplicates will be expected. All screening decisions will follow predefined inclusion and exclusion criteria. Two researchers will independently conduct the screening. Blinding of reviewers will not be implemented, as the intervention names and regulatory context are inherently associated with eligibility decisions. All screening decisions will be documented to promote transparency and consistency.

Studies will be excluded if they: (1) do not involve DiGAs or digital health interventions within the German context; (2) do not report the use of single-item scales (e.g., those that rely solely on multi-item measures); or (3) focus on non-healthcare outcomes. Additionally, non-primary research (reviews, commentaries, protocols, editorials, and opinion pieces) will be excluded, as will any publications not available in English or German (Table 1).

**Table 1:** Eligibility criteria based on the Population–Concept–Context (PCC) framework and additional study selection criteria.

PCC- Component	Inclusion Criteria	Exclusion Criteria
Population	DiGAs listed in the BfArM DiGA directory	Digital health interventions not classified as DiGAs or not within the German reimbursement system
Concept	Studies reporting the use of single-item scales in DiGA evaluations, including their application, constructs, or reported methodological considerations	Studies not using single-item measures or relying exclusively on multi-item instruments
Context	Evaluations conducted within the German healthcare system, including DiGA fast-track or reimbursement-related studies	Studies outside the German healthcare context or not linked to DiGA evaluation processes
**Methodological Components**	**Inclusion Citeria**	**Exclusion Criteria**
Study type / design	Primary empirical studies	Reviews, commentaries, protocols, editorials, or opinion pieces that do not provide primary data.
Outcome Scope	Studies reporting healthcare-related outcomes relevant to the evaluation of DiGAs	Studies focused on non-healthcare outcomes (e.g., project management, IT trust, software usability unrelated to health)
Language	Publications available in English or German	Publications in languages other than English or German

### Managing overlapping and duplicate publications

As studies will be identified through the BfArM DiGA directory, multiple publications reporting on the same DiGA evaluation may exist (e.g., different outcomes, follow-up periods, or subgroup analyses). To address this, all publications linked to a single DiGA will be grouped at the DiGA level, which will serve as the primary unit of analysis. Where multiple publications report on the same underlying study or patient cohort, information will be extracted from all relevant sources to ensure completeness, while avoiding double counting in descriptive summaries. Duplicate or overlapping records will be identified through comparison of trial registrations, DiGA names, study titles, and author information, and resolved through discussion between reviewers. Data will be consolidated at the DiGA level.

### Data charting (extraction and data management)

Data extraction will follow a predefined framework. The extracted variables are organized into four domains: (1) source and bibliographic information, (2) study characteristics, (3) intervention and DiGA characteristics, and (4) outcome and methodological information (Table 2).

**Table 2:** Data items to be collected.

Category	Data
Source Details / Bibliographic Information	• Author(s) •Year of publication •Journal or publication outlet • Country • Funding source (if reported) • DOI or URL
Study Characteristics	• Study design (e.g., RCT, non-randomized, observational) • Evaluation context (routine care, field-based, clinical) • Sample size • Target population (age, condition, patient group) • Comparator(s), if applicable
Intervention / Digital Health Application	• DiGA name • Therapeutic area / indication • Intended purpose (e.g., treatment support, monitoring, prevention) • Evaluation phase (e.g., provisional, permanent listing)
Outcome / Concept Measured	• Use of single-item scale(s) (yes/no) • Constructs assessed (e.g., well-being, quality of life, satisfaction) • Wording of the single-item scale(s), if available • Response format (e.g., numeric rating scale, Likert scale)
Results / Findings / Methodological Rationale	• Authors’ rationale for using single-item scale(s) • Reported advantages of single-item scale use • Reported limitations or concerns • Psychometric considerations (validity, reliability, sensitivity to change, if reported) • Role in demonstrating positive healthcare effect • Authors’ reflections on feasibility, participant burden, and real-world applicability • Methodological gaps or recommendations noted by authors

Data extraction will be conducted by two independent researchers. First, a pilot extraction phase will be performed by both researchers on a small subset of included sources to calibrate the extraction form and ensure a shared understanding of extraction categories. Second, the main extraction will be conducted independently and in parallel by both researchers using a structured spreadsheet. Third, the two independently extracted datasets will be compared, and any disagreements will be documented in the extraction template and resolved through consensus. Data extraction will be managed using structured spreadsheets, while study selection and screening will be conducted in the CADIMA web-based platform.

In accordance with recommendations from the Joanna Briggs Institute for transparent and reproducible scoping reviews, all extracted data will be recorded in structured spreadsheet files (XLSX/ODS) and stored in the OSF. These files will provide a complete record of all variables extracted from each included source. The materials will be made publicly available upon completion of the review, except where copyright restrictions apply.

### Collating, summarizing, and reporting results

To address the research questions, extracted data will be collated and synthesized using a descriptive mapping approach. Quantitative information (e.g., number of DiGAs using single-item scales, sample sizes, and study characteristics) will be summarized using descriptive statistics and presented in structured tables to provide an exploratory mapping rather than a confirmatory analysis. Quantitative synthesis in the form of meta-analysis is not planned and no criteria for statistical pooling of results are defined, as the scoping review aims to provide a descriptive mapping rather than to estimate pooled effects. Qualitative information, including constructs assessed, reported rationales, advantages, limitations, and methodological considerations, will be thematically categorized to identify patterns and gaps across studies. Data will not be transformed beyond minor standardization of units where necessary to ensure comparability and clarity of presentation. Missing or incomplete data will be documented and, where feasible, supplemented through contact with study authors. The inclusion of DiGAs across diverse clinical domains may introduce contextual heterogeneity; however, this is not the focus of analysis.

To ensure data accuracy and validity, data extracted independently by both researchers will be compared and reconciled through discussion to achieve consensus. The agreed-upon dataset will then be cross-checked against the BfArM DiGA directory and corresponding publications to ensure the completeness and accuracy of extracted study information, while bibliographic details will be triangulated using PubMed records where necessary to verify publication metadata. Any unclear or inconsistent information will be documented and resolved through discussion, with all decisions recorded to ensure transparency. In line with scoping review methodology, no formal critical appraisal of study quality or risk of bias will be conducted, and the strength of the body of evidence will not be assessed (e.g., GRADE), as the aim is to map and characterize the use of single-item scales rather than to evaluate the effectiveness or certainty of evidence.

The synthesis will be conducted by the primary researcher and reviewed by a second researcher to enhance reliability and consistency of categorization and interpretation within the exploratory scope of this scoping review. Results will be presented in a structured and transparent manner using tables and figures that link DiGA characteristics, measurement practices, and reported outcomes. These will be complemented by a narrative summary to contextualize the findings and to highlight observed methodological patterns, potential evidence gaps, and preliminary implications for outcome measurement in regulated digital health applications.

For this scoping review, the primary outcome is the characterization of the use of single-item scales in the evaluation of DiGAs, including the frequency and way these measures are applied across DiGA evaluations.

Additional outcomes include the constructs assessed using single-item scales (e.g., well-being, quality of life, satisfaction), the reported rationales for their use, and the reported advantages and limitations associated with single-item measurement approaches. Further outcomes comprise methodological considerations reported in the literature, including psychometric properties (e.g., validity, reliability, sensitivity to change where available), as well as authors’ reflections on feasibility, participant burden, real-world applicability, and the role of single-item measures in demonstrating positive healthcare effects.

No formal hierarchy beyond this distinction between primary and additional outcomes is predefined, and all outcomes will be analyzed within a descriptive and thematic framework.

### Consultation

As part of the scoping review framework, a consultation stage is planned to enhance the relevance and applicability of the findings. Following the initial synthesis, the preliminary results will be discussed with stakeholders, including researchers with expertise in DiGA evaluation and measurement methodology (e.g., through national conferences). This consultation aims to contextualize the findings, validate the interpretation of patterns and gaps, and identify practical implications for outcome measurement in DiGA evaluations. Feedback received will be integrated into the final presentation of results. The consultation stage is scheduled for 2027.

### Study status and timeline

The scoping review is currently at the protocol stage and has not yet commenced; the start of the review process is scheduled for June 2026. Study selection and data extraction are expected to be completed by December 2026, reflecting the two-stage screening and dual-reviewer verification process. Data synthesis is expected to be finalized by May 2027. The results of the review are expected to be available by June 2027. As this study does not involve human participants, participant recruitment is not applicable.

### Ethics and dissemination

Ethical approval will not be required for this paper, as it will rely on database entries and already published articles.

## Supporting information

S1 File PRISMA-P (Preferred Reporting Items for Systematic review and Meta-Analysis Protocols) 2015 Checklist. (DOCX)
