# Supplementary material for: Use of single-item scales in the evaluation of German digital health applications (DiGAs): Scoping review protocol
Source: PLoS One. 2026 Aug 26;21(8):e0356485. doi: 10.1371/journal.pone.0356485 (PMC13514044; doi:10.1371/journal.pone.0356485)
Supplement: Supplementary file 2 [file pone.0356485.s002.docx]

**Reviewer Comments**

*Use of Single-Item Scales in the Evaluation of German Digital Health Applications (DiGAs): Scoping Review Protocol*

Thank you for sharing this protocol. The topic is relevant, the literature base is appropriate, and the motivation for the review is clear. My comments below are intended to strengthen the manuscript.

**ABSTRACT**

**1. Introduction sentence needs rewriting:** The opening of the abstract is confusing as currently written. Terms like "reimbursable" are used without sufficient context for a general reader and the core message is unclear. Recommend rewriting to better reflect and communicate what is in the introduction.

**2. Language implies a systematic rather than scoping review:** Bold claims in the abstract suggest a systematic examination of evidence, which overstates the intent of a scoping review. Scoping reviews are exploratory in nature and the language should reflect that.

**INTRODUCTION**

**3. Sentences are too long** Several sentences in the introduction are unwieldy and need to be shortened for readability.

**4. Single-item scale measurement is not defined:** The introduction does not explain or illustrate what a single-item scale actually is. Recommend defining it clearly and directing readers to the mini-narrative review by Lauren Staples: *"Single item measures in digital mental healthcare: a mini narrative review of challenges and opportunities."*

**5. Introduction is biased toward disadvantages:** The introduction leans heavily toward the limitations of single-item scales, which mirrors the apparent direction of the review. To present a balanced foundation, dedicate one paragraph to the advantages of single-item scales and a separate paragraph to the challenges.

**6. Objectives are buried in prose:** The objectives are lost within paragraphs rather than stated clearly and directly. They should be presented in a simple, explicit, standalone format.

**7. The scope is too broad — specify the clinical domain:** Digital health spans many fields (cardiology, psychiatry, oncology, etc.) and the constructs being measured will differ substantially across them. Mental health differs meaningfully from, say, cardiology, and this distinction matters for both the aims and the search strategy.

**METHODS**

**8. Rewrite using Arksey and O'Malley's six-stage framework as subheadings:** The current methods section is repetitive and omits key information. The entire section should be restructured around Arksey's framework, using the following as subheadings:

- Formulation of the research question
- Identifying relevant studies
- Selecting eligible studies (inclusion and exclusion criteria)
- Data charting (extraction and data management)
- Collating, summarising, and reporting results
- Consultation

**9. Exclusion criteria are missing:** The current version discusses inclusion criteria but omits exclusion criteria entirely. Both must be included under the study selection section.

**10. Revise the PCC framework:** The Population, Concept, and Context (PCC) framework needs to be revisited and revised. Inclusion and exclusion criteria should each be presented as a subsection with the PCC framework embedded.

**11. Critical appraisal section is absent:** There is no mention of whether critical appraisal will be conducted. A dedicated section should address this explicitly — whether appraisal will be done, and if not, why not, which is acceptable in a scoping review but must be stated.

**12. No section on managing overlapping publications** :A section addressing how overlapping or duplicate publications will be identified and handled is missing.

**13. Search strategy needs revision:** The current strategy searches for DiGAs broadly rather than specifically for single-item scale measurement *within* DiGA evaluations. This needs to be refocused. Additional concerns:

- The current approach risks producing homogeneous, grey-literature-heavy results
- A librarian should be engaged to access multiple databases systematically
- Be explicit about whether sources are published literature or grey literature
- The search strategy should be included as an appendix
- Sources for screening should be more diverse

**14. Explain what the DiGA directory contains:** It is not clear what type of information is held in the DiGA directory, how many studies have evaluated DiGAs, or how many of those used single-item scales. This context is needed.

**15. Data extraction section is poorly organised:** The data extraction section is fragmented and needs to be reorganized clearly, specifying what data will be extracted and how it will be managed.

**16. Screening should involve two independent reviewers:** Screening and extraction should be performed concurrently by two independent researchers who then compare and reconcile their work. Having one researcher verify the work of another introduces bias and is not standard practice.

**17. Specify whether a screening tool will be used:** It should be stated whether a tool such as Covidence will be used to manage screening and extraction.

**OVERALL / CROSS-CUTTING**

**18. The central focus of the review is unclear:** It is not consistently clear whether the review is about DiGAs themselves or about the *use of single-item scales in the evaluation of DiGAs*. If it is the latter — which the title suggests — the entire manuscript needs to be adjusted to reflect this focus consistently throughout.

**19. Reference 2 is a conference abstract:** Fujita-Rohwerder and Sauerland (2022) is a conference abstract, not a full peer-reviewed publication. This should be acknowledged, or a fuller publication substituted if available.

**20. Reduce repetition throughout the manuscript:** The same content appears in multiple sections. A thorough edit for redundancy across the abstract, introduction, and methods is needed.
