# Supplementary material for: Use of single-item scales in the evaluation of German digital health applications (DiGAs): Scoping review protocol
Source: PLoS One. 2026 Aug 26;21(8):e0356485. doi: 10.1371/journal.pone.0356485 (PMC13514044; doi:10.1371/journal.pone.0356485)
Supplement: Supplementary file 3 [file pone.0356485.s004.docx]

Dear academic editor and reviewer,

We sincerely thank you for the time and effort you invested in reviewing our manuscript entitled " Use of single-item scales in the evaluation of German digital health applications (DiGAs): Scoping review protocol" We greatly appreciate the constructive and detailed comments, which have helped us to improve the clarity, methodological transparency, and overall quality of the manuscript.

We have carefully considered every comment and revised the manuscript accordingly. In the point-by-point responses below, each reviewer comment is reproduced in bold, followed by our response and, where appropriate, verbatim excerpts from the revised manuscript to facilitate review. All changes in the revised manuscript have been highlighted using track changes.

We hope that the revisions satisfactorily address all concerns and that the revised manuscript is now suitable for publication. We thank you again for your time and thoughtful feedback and for the opportunity to improve our work.

*Sincerely,*

*the authors*

**1-Editor Comment:**

Please ensure that your manuscript meets PLOS ONE's style requirements, including file naming.

**Answer:**

We confirm that the revised manuscript has been checked for compliance with the PLOS ONE formatting and style requirements, including manuscript formatting and file naming, in accordance with the journal guidelines.

**2 & 3-Editor Comments:**

Please remove funding information from the manuscript and provide the revised Funding Statement for the online submission system.

**Answer:**

We thank the Editorial Office for these clarifications regarding the Funding Statement and Financial Disclosure.

In accordance with the Academic Editor's instructions, we removed all funding-related information from the manuscript. At that stage, we did not modify the online submission system, as the decision letter explicitly instructed us to provide the revised statements in our cover letter and indicated that the journal would update the submission metadata on our behalf.

Following the subsequent editorial request indicating that additional changes to the Funding Information and Financial Disclosure were required, we have now updated the information in the online submission system ourselves to ensure consistency between both sections and to avoid further delays in the review process.

The consolidated Financial Disclosure statement now reads:

*Open Access publication was supported by IU International University of Applied Sciences (https://www.iu.org). No research grant was awarded for this study; therefore, no grant number exists. Author JW received Open Access publication support and is employed by IU International University of Applied Sciences. The funder provided support in the form of salaries for author JW, but did not have any additional role in the study design, data collection and analysis, decision to publish, or preparation of the manuscript. The specific role of this author is articulated in the "Contributions" section.*

**4-Editor Comment:**

Clarify the role of the funding organization by confirming that its involvement was limited to financial support (author salary and/or publication costs), revise the Author Contributions accordingly, and provide the requested Financial Disclosure statement.

**Answer:**

4.1 We thank the Editorial Office for this clarification. We have revised the Author Contributions to provide a more transparent and standardized description of each author's contributions. The revised Author Contributions state:

*“JW conceived the study, developed the methodological approach, prepared the original draft of the protocol, and led the manuscript revision, including reviewing and integrating revisions prior to submission. MM contributed to the methodological development of the protocol and provided critical intellectual feedback through review and editing of the manuscript.”*

4.2 We thank the Editorial Office for this clarification.

In response to the Academic Editor's previous instructions, we revised the Author Contributions to provide a more transparent and standardized description of each author's role in accordance with the CRediT (Contributor Roles Taxonomy). At that stage, we also confirmed our agreement with the requested Financial Disclosure statement in the cover letter, as the decision letter instructed us not to modify the online submission system and indicated that the journal would update the submission metadata on our behalf.

Following the subsequent editorial request for additional revisions to the Funding Information and Financial Disclosure, we have now updated the online submission system ourselves to ensure that all funding and employment disclosures are presented consistently in a single Financial Disclosure statement.

Accordingly, the revised Financial Disclosure includes the wording:

*"The funder provided support in the form of salaries for author JW, but did not have any additional role in the study design, data collection and analysis, decision to publish, or preparation of the manuscript. The specific role of this author is articulated in the "Contributions" section."*

The Author Contributions remain unchanged from our previous revision, as they already accurately describe the specific roles of each author.

**5-Editor Comment:**

If the reviewer comments include a recommendation to cite specific previously published works, please review and evaluate these publications to determine whether they are relevant and should be cited.

**Answer:**

We carefully evaluated all references suggested by the reviewer. Relevant literature has been incorporated into the revised manuscript where appropriate. References that were not considered directly relevant to the objectives and scope of this protocol were not included.

**1-Reviewer Comment:**

Introduction sentence needs rewriting: The opening of the abstract is confusing as currently written. Terms like “reimbursable” are used without sufficient context for a general reader and the core message is unclear. Recommend rewriting to better reflect and communicate what is in the introduction.

**Answer**:

We thank the reviewer for this helpful suggestion. We agree that the opening sentence of the abstract required additional context for readers who may be unfamiliar with the German DiGA framework. We have therefore revised the opening of the introduction to introduce DiGAs more clearly and to explain their reimbursement within the German healthcare system. The revised wording provides the necessary context before introducing the methodological focus of the review.

**Revised manuscript (Abstract):**

*"Digital health applications are increasingly integrated into routine healthcare delivery. In Germany, so-called Digitale Gesundheitsanwendungen (DiGAs) represent a distinct category of patient-facing digital health applications. These applications can be prescribed*

*by medical doctors and are reimbursed by statutory health insurance schemes once they are listed in the official DiGA directory maintained by the German Federal Institute for Drugs and Medical Devices (BfArM) [1]. The DiGA directory is a regulatory register of all Digital Health Applications that have successfully completed the statutory assessment procedure under §139e SGB V and the German Digital Health Applications Ordinance (DiGAV). It provides structured information on each DiGA, including basic product characteristics (e.g., name, manufacturer, intended medical purpose), functional description, usage conditions, data protection and interoperability features, and information relevant to healthcare providers and patients. Importantly, it also contains references to the scientific evidence submitted for reimbursement approval, including evaluation studies and trial registrations used to demonstrate positive healthcare effects."*

**2-Reviewer Comment:**

Language implies a systematic rather than scoping review: Bold claims in the abstract suggest a systematic examination of evidence, which overstates the intent of a scoping review. Scoping reviews are exploratory in nature and the language should reflect that.

**Answer:**

We thank the reviewer for this important methodological observation. We agree that the language used throughout the manuscript should accurately reflect the exploratory nature of a scoping review.

In response, we carefully reviewed the manuscript and revised the wording where appropriate to ensure consistency with scoping review methodology. In particular, the review is now consistently presented as a **descriptive mapping** of the use of single-item scales in DiGA evaluations rather than as a systematic synthesis of evidence. The revised manuscript also explicitly states that quantitative findings will be summarized descriptively, that no meta-analysis is planned, and that no formal critical appraisal of included studies will be undertaken, in accordance with established scoping review methodology.

**3-Reviewer Comment:**

Sentences are too long Several sentences in the introduction are unwieldy and need to be shortened for readability.

**Answer:**

We thank the reviewer for this helpful comment. We carefully revised the Introduction to improve readability by shortening several overly long sentences and simplifying the overall structure where appropriate. In addition, we removed redundant wording and reorganized parts of the introduction into shorter, more focused paragraphs. These revisions improve the flow of the text while preserving the intended content and level of detail.

**4-Reviewer Comment:**

Single-item scale measurement is not defined: The introduction does not explain or illustrate what a single-item scale actually is. Recommend defining it clearly and directing readers to the mini-narrative review by Lauren Staples: "Single item measures in digital mental healthcare: a mini narrative review of challenges and opportunities."

**Answer:**

We thank the reviewer for this helpful suggestion. We agree that the introduction should clearly define the concept of a single-item scale before discussing its application in DiGA evaluations.

In response, we expanded the Introduction by adding a definition of single-item scales and by providing additional context on their use in digital health interventions. We also incorporated the recommended mini-narrative review by Staples et al. (2026) to contextualize the use of single-item measures in digital mental healthcare.

**Revised manuscript (Introduction):**

*"* *A single-item scale is a measurement instrument consisting of only one question or statement to assess a particular construct or attribute. Despite their simplicity, these scales can be effective for measuring various aspects, such as symptoms, satisfaction, or perceived health status, particularly in contexts where brevity is essential, such as in digital health intervention [5–7]. For example, a commonly used single-item measure is the global health question: "In general, would you say your health is excellent, very good, good, fair, or poor?" [5]. Recent evidence from digital mental health contexts further supports the practical utility of single-item scales for large-scale screening, outcome monitoring, and integration into digital platforms. This review provides a valuable synthesis of single-item scales use in digital mental healthcare from a clinical effectiveness perspective [8]."*

**5-Reviewer Comment:**

Introduction is biased toward disadvantages: The introduction leans heavily toward the limitations of single-item scales, which mirrors the apparent direction of the review. To present a balanced foundation, dedicate one paragraph to the advantages of single-item scales and a separate paragraph to the challenges.

**Answer:**

We thank the reviewer for this valuable suggestion. We agree that the introduction should provide a balanced overview of both the advantages and limitations of single-item scales before introducing the rationale for the review.

In response, we revised the Introduction by separating the discussion into two distinct paragraphs. The first paragraph summarizes the practical and methodological advantages of single-item scales, including their suitability for digital health interventions, reduced response burden, and evidence supporting their use for assessing clearly defined constructs. The subsequent paragraph discusses their methodological limitations, including restricted construct coverage, the inability to assess internal consistency, and the importance of careful theoretical justification.

This revised structure provides a more balanced and neutral introduction to the topic and better reflects the exploratory nature of the proposed scoping review.

**Revised manuscript (Introduction):**

*"In this context, single-item scales offer a promising approach to mitigating competing demands between methodological rigor and feasibility. A single-item scale is a measurement instrument consisting of only one question or statement to assess a particular construct or attribute. Despite their simplicity, these scales can be effective for measuring various aspects, such as symptoms, satisfaction, or perceived health status, particularly in contexts where brevity is essential, such as in digital health intervention [5–7]. For example, a commonly used single-item measure is the global health question: "In general, would you say your health is excellent, very good, good, fair, or poor?" [5]. Recent evidence from digital mental health contexts further supports the practical utility of single-item scales for large-scale screening, outcome monitoring, and integration into digital platforms. This review provides a valuable synthesis of single-item scales use in digital mental healthcare from a clinical effectiveness perspective [8]. More recent clinical research further supports the utility of single-item quality-of-life assessments, demonstrating robust associations with well-being, health indicators, and lifestyle-related correlates [6]. Methodological studies further suggest that single-item measures may be appropriate when constructs are unidimensional, concrete, and easily understood by respondents, particularly when minimizing participant burden is a priority [7]."*

**6-Reviewer Comment:**

Objectives are buried in prose: The objectives are lost within paragraphs rather than stated clearly and directly. They should be presented in a simple, explicit, standalone format.

**Answer:**

We thank the reviewer for this helpful suggestion. We agree that the objectives should be presented in a clear and easily identifiable format.

In response, we restructured the **Aims and objectives** section by separating the overall aim from the specific objectives and presenting the latter as a standalone list. We also added a separate **Expected Outcomes** subsection to distinguish the objectives of the review from the anticipated contributions of the study.

**Revised manuscript (Aims and objectives – Objectives):**

• *To identify which DiGA evaluations employ single-item scales.*

*• To describe the constructs assessed using single-item scales.*

*• To map the reported rationales, advantages, and limitations associated with their use.*

*• To identify methodological patterns and gaps in current evaluation practices.*

**7-Reviewer Comment:**

The scope is too broad — specify the clinical domain: Digital health spans many fields (cardiology, psychiatry, oncology, etc.) and the constructs being measured will differ substantially across them. Mental health differs meaningfully from, say, cardiology, and this distinction matters for both the aims and the search strategy.

**Answer:**

We thank the reviewer for this thoughtful comment. We agree that Digital Health Applications encompass a broad range of clinical indications and that the constructs assessed may vary across therapeutic areas.

However, the objective of this scoping review is methodological rather than clinical. The review does not aim to compare measurement practices between disease areas; instead, it seeks to map the use of single-item scales across DiGA evaluations within the German regulatory framework. Consequently, the use of single-item measures constitutes the primary unit of analysis, whereas the clinical indication of a DiGA serves only as contextual information.

To clarify this rationale, we have revised the manuscript by explicitly stating that clinical indications are not used as analytical strata because the review focuses on measurement methodology across DiGAs as a unified regulatory sampling frame.

**Revised manuscript (Identifying the research question):**

*"Clinical indications of DiGAs (e.g., psychiatry, cardiology, dermatology) are not used as analytical strata in this review, as the focus is exclusively on measurement methodology, specifically the use of single-item scales, across DiGAs as a unified regulatory sampling frame within the German reimbursement system, while the use of single-item measures constitutes the primary unit of analysis and the central focus of this scoping review.*

**8-Reviewer Comment:**

Rewrite using Arksey and O'Malley's six-stage framework as subheadings: The current methods section is repetitive and omits key information. The entire section should be restructured around Arksey's framework, using the following as subheadings:

•	Formulation of the research question

•	Identifying relevant studies

•	Selecting eligible studies (inclusion and exclusion criteria)

•	Data charting (extraction and data management)

•	Collating, summarising, and reporting results

•	Consultation

**Answer:**

We thank the reviewer for this constructive suggestion. We agree that structuring the Methods section according to the Arksey and O'Malley framework improves clarity and better reflects the methodological approach of the review.

In response, we comprehensively reorganized the **Methods and Analysis** section using the six stages of the Arksey and O'Malley framework as the primary subheadings. During this revision, we also consolidated overlapping content and relocated methodological information to the appropriate sections to improve the logical flow and reduce redundancy.

**The revised manuscript is now structured according to the following stages:**

- *Identifying the research question*
- *Identifying relevant studies*
- *Selecting eligible studies (inclusion and exclusion criteria)*
- *Data charting (extraction and data management)*
- *Collating, summarizing, and reporting results*
- *Consultation*

**9-Reviewer Comment:**

Exclusion criteria are missing: The current version discusses inclusion criteria but omits exclusion criteria entirely. Both must be included under the study selection section.

**Answer:**

We thank the reviewer for this helpful comment. While exclusion criteria were included in the original manuscript, we agree that they could have been presented more clearly within the study selection process.

In response, we revised the **Selecting eligible studies (inclusion and exclusion criteria)** subsection to explicitly describe both the inclusion and exclusion criteria. We also revised **Table 1** to present these criteria in a clearer, structured format based on the Population–Concept–Context (PCC) framework, thereby improving the transparency of the eligibility and screening process.

**10-Reviewer Comment:**

Revise the PCC framework: The Population, Concept, and Context (PCC) framework needs to be revisited and revised. Inclusion and exclusion criteria should each be presented as a subsection with the PCC framework embedded.

**Answer:**

We thank the reviewer for this helpful suggestion. We agree that the role of the Population–Concept–Context (PCC) framework should be more clearly integrated into the methodological description of the review.

In response, we revised the manuscript to introduce the PCC framework directly within the **Identifying the research question** subsection, where it is explicitly linked to the development of the research question and the eligibility criteria. We also revised **Table 1** to present the eligibility criteria in a structured format based on the PCC framework, together with additional methodological inclusion and exclusion criteria. These revisions clarify how the PCC framework informs the study selection process.

**Revised manuscript (Identifying the research question):**

*"This question was developed using the Population–Concept–Context (PCC) framework [13], which serves as the foundation for our eligibility criteria:"*

**11-Reviewer Comment:**

Critical appraisal section is absent: There is no mention of whether critical appraisal will be conducted. A dedicated section should address this explicitly — whether appraisal will be done, and if not, why not, which is acceptable in a scoping review but must be stated.

**Answer:**

We thank the reviewer for this important methodological comment. We agree that the manuscript should explicitly state whether a formal critical appraisal will be conducted and provide the rationale for this decision.

Although the original manuscript indicated that no formal critical appraisal was planned, we have expanded this section to provide a clearer methodological justification. Specifically, we now explicitly state that neither a formal critical appraisal nor an assessment of the overall strength of the evidence (e.g., GRADE) will be undertaken, as the objective of this scoping review is to map and characterize the use of single-item scales rather than to evaluate intervention effectiveness or the certainty of the available evidence.

**Revised manuscript (Collating, summarizing, and reporting results):**

*"In line with scoping review methodology, no formal critical appraisal of study quality or risk of bias will be conducted, and the strength of the body of evidence will not be as-sessed (e.g., GRADE), as the aim is to map and characterize the use of single-item scales rather than to evaluate the effectiveness or certainty of evidence.*

**12-Reviewer Comment:**

No section on managing overlapping publications: A section addressing how overlapping or duplicate publications will be identified and handled is missing.

**Answer:**

We thank the reviewer for this important methodological suggestion. In response, we added a dedicated subsection entitled **"Managing overlapping and duplicate publications"** to describe how multiple publications arising from the same DiGA evaluation will be identified and handled.

The revised section explains that the DiGA serves as the primary unit of analysis, that publications reporting on the same underlying study or patient cohort will be identified through comparison of trial registrations, DiGA names, study titles, and author information, and that information from overlapping reports will be consolidated while avoiding double counting in descriptive summaries.

**Revised manuscript (Managing overlapping and duplicate publications):**

*"* *As studies will be identified through the BfArM DiGA directory, multiple publications re-porting on the same DiGA evaluation may exist (e.g., different outcomes, follow-up peri-ods, or subgroup analyses). To address this, all publications linked to a single DiGA will be grouped at the DiGA level, which will serve as the primary unit of analysis. Where mul-tiple publications report on the same underlying study or patient cohort, information will be extracted from all relevant sources to ensure completeness, while avoiding double counting in descriptive summaries. Duplicate or overlapping records will be identified through comparison of trial registrations, DiGA names, study titles, and author infor-mation, and resolved through discussion between reviewers. Data will be consolidated at the DiGA level."*

**13-Reviewer Comment:**

Search strategy needs revision: The current strategy searches for DiGAs broadly rather than specifically for single-item scale measurement within DiGA evaluations. This needs to be refocused. Additional concerns:

•	The current approach risks producing homogeneous, grey-literature-heavy results

•	A librarian should be engaged to access multiple databases systematically

•	Be explicit about whether sources are published literature or grey literature

•	The search strategy should be included as an appendix

•	Sources for screening should be more diverse

**Answer:**

We thank the reviewer for this thoughtful methodological comment. We agree that the search strategy should clearly reflect the objectives of the review and transparently describe the sources of evidence.

However, the primary objective of this scoping review is to map the use of single-item scales across **all reimbursable German Digital Health Applications (DiGAs)**. Accordingly, the review uses the official BfArM DiGA directory as a registry-based sampling frame rather than a conventional keyword-based literature search. This approach ensures comprehensive identification of all DiGA evaluations considered within the German reimbursement process and minimizes the risk of missing relevant studies in which the use of single-item measures is not explicitly reported in titles, abstracts, or indexing terms.

To clarify this methodology, we substantially expanded the **Identifying relevant studies** subsection. The revised manuscript now explains the contents of the DiGA directory, specifies that linked evaluation studies and trial registrations constitute the primary evidence sources, clarifies that PubMed is used only to verify or complete bibliographic information where necessary, and states that all search and verification procedures will be systematically documented to ensure transparency and reproducibility.

**Revised manuscript (Identifying relevant studies):**

*"* *The primary information source for this scoping review will be the official DiGA directory maintained by the German Federal Institute for Drugs and Medical Devices (BfArM), which provides a comprehensive and authoritative listing of all reimbursable Digital Health Applications in Germany. As this directory constitutes the complete regulatory register of DiGAs, all DiGAs listed in the directory will be included."*

**14-Reviewer Comment:**

Explain what the DiGA directory contains: It is not clear what type of information is held in the DiGA directory, how many studies have evaluated DiGAs, or how many of those used single-item scales. This context is needed.

**Answer:**

We thank the reviewer for this valuable suggestion. We agree that the role and contents of the DiGA directory should be explained more clearly to justify its use as the primary information source for this scoping review.

In response, we substantially expanded the Introduction and the **Identifying relevant studies** subsection to describe the contents of the BfArM DiGA directory. The revised manuscript now explains that the directory provides structured information on each DiGA, including product characteristics, regulatory information, and, importantly, references to the scientific evaluation studies and trial registrations submitted as part of the reimbursement process. This clarification provides the rationale for using the DiGA directory as the regulatory sampling frame for the review.

As this manuscript describes a **study protocol**, the number of evaluation studies and the number of studies using single-item scales cannot yet be reported, as these are outcomes of the planned review rather than information available at the protocol stage.

**Revised manuscript (Introduction):**

*"The DiGA directory is a regulatory register of all Digital Health Applications that have suc-cessfully completed the statutory assessment procedure under §139e SGB V and the German Digital Health Applications Ordinance (DiGAV). It provides structured infor-mation on each DiGA, including basic product characteristics (e.g., name, manufacturer, intended medical purpose), functional description, usage conditions, data protection and interoperability features, and information relevant to healthcare providers and pa-tients. Importantly, it also contains references to the scientific evidence submitted for reimbursement approval, including evaluation studies and trial registrations used to demonstrate positive healthcare effects."*

**15-Reviewer Comment:**

Data extraction section is poorly organised: The data extraction section is fragmented and needs to be reorganized clearly, specifying what data will be extracted and how it will be managed.

**Answer:**

We thank the reviewer for this valuable suggestion. We agree that the data extraction procedures should be presented in a clearer and more structured manner.

In response, we comprehensively reorganized the **Data charting (extraction and data management)** subsection. The revised manuscript now clearly distinguishes between the extraction framework, the extraction procedure, data management, and the variables to be collected. Specifically, we describe the four domains of extracted information, outline the pilot and independent extraction process undertaken by two reviewers, explain how disagreements will be resolved, and specify how extracted data will be managed and made publicly available. In addition, **Table 2** was aligned with this revised structure to provide a clear overview of all data items to be collected. These revisions improve the transparency and reproducibility of the planned data charting process.

**16-Reviewer Comment:**

Screening should involve two independent reviewers: Screening and extraction should be performed concurrently by two independent researchers who then compare and reconcile their work. Having one researcher verify the work of another introduces bias and is not standard practice.

**Answer:**

We thank the reviewer for this important methodological comment. We agree that independent screening and data extraction by two reviewers enhances methodological rigor and reduces the potential for bias.

In response, we revised the manuscript to clarify that both study screening and data extraction will be conducted independently by two researchers. For data extraction, the revised protocol now specifies a pilot extraction phase followed by independent and parallel extraction by both reviewers. The independently extracted datasets will then be compared, with any disagreements documented and resolved through consensus. This revision replaces the previous verification approach and aligns the protocol with established methodological guidance for scoping reviews.

**Revised manuscript (Data charting (extraction and data management)):**

*"Data extraction will be conducted by two independent researchers. First, a pilot extrac-tion phase will be performed by both researchers on a small subset of included sources to calibrate the extraction form and ensure a shared understanding of extraction catego-ries. Second, the main extraction will be conducted independently and in parallel by both researchers using a structured spreadsheet. Third, the two independently extracted da-tasets will be compared, and any disagreements will be documented in the extraction template and resolved through consensus."*

**17-Reviewer Comment:**

Specify whether a screening tool will be used: It should be stated whether a tool such as Covidence will be used to manage screening and extraction.

**Answer:**

We thank the reviewer for this helpful suggestion. We agree that the manuscript should clearly specify the tools used to manage the review process.

In response, we clarified the software used for study management throughout the protocol. The revised manuscript explicitly states that the **CADIMA** web-based platform will be used to manage study selection, screening, and documentation of the review process, while data extraction will be conducted using structured spreadsheet files. Although the reviewer mentioned Covidence as an example, we elected to use CADIMA because it provides the required functionality for the planned scoping review.

**Revised manuscript (Identifying relevant studies):**

*"Finally, all records will be transferred to the CADIMA platform, which will be used to manage study selection, screening, and documentation of the review process."*

**18-Reviewer Comment:**

The central focus of the review is unclear: It is not consistently clear whether the review is about DiGAs themselves or about the *use of single-item scales in the evaluation of DiGAs*. If it is the latter — which the title suggests — the entire manuscript needs to be adjusted to reflect this focus consistently throughout.

**Answer:**

We thank the reviewer for this important observation. We agree that the central methodological focus of the review should be communicated consistently throughout the manuscript.

In response, we carefully revised the manuscript to emphasize that the primary focus of this scoping review is the **use of single-item scales in the evaluation of German Digital Health Applications (DiGAs)**, rather than the DiGAs themselves. Accordingly, the Introduction, Aims and Objectives, and Methods sections were revised to consistently frame DiGAs as the regulatory sampling frame and single-item scales as the primary methodological focus of the review.

**Revised manuscript (Introduction):**

*"Despite evidence on both advantages and limitations in health-related research, the use of single-item scales in DiGA evaluations has not been systematically mapped. It remains unclear which constructs are assessed using single-item measures and how their use is justified within this regulated reimbursement context. This scoping review addresses this gap by systematically mapping single-item scale use across DiGA evaluation studies."*

**19-Reviewer Comment:**

Reference 2 is a conference abstract: Fujita-Rohwerder and Sauerland (2022) is a conference abstract, not a full peer-reviewed publication. This should be acknowledged, or a fuller publication substituted if available.

**Answer:**

We thank the reviewer for this observation. We acknowledge that the publication by Fujita-Rohwerder and Sauerland is a conference abstract rather than a full peer-reviewed article.

We reviewed the available literature to determine whether a corresponding full-length publication was available but did not identify one. We therefore retained this reference because it remains one of the few published sources specifically addressing evidence generation within the German DiGA reimbursement process. In the revised manuscript, the conference abstract is used only to provide supplementary background information, whereas the regulatory framework and methodological aspects of the protocol are primarily supported by official BfArM documentation and additional peer-reviewed literature.

Furthermore, we note that scoping reviews generally adopt a comprehensive approach to identifying and reporting relevant sources of evidence, including grey literature where appropriate. While the conference abstract is not part of the planned evidence synthesis, we consider it an appropriate contextual reference in the absence of a corresponding full-length publication.

**20-Reviewer Comment:**

Reduce repetition throughout the manuscript: The same content appears in multiple sections. A thorough edit for redundancy across the abstract, introduction, and methods is needed.

**Answer:**

We thank the reviewer for this valuable comment. We agree that reducing redundancy improves the clarity and readability of the manuscript.

In response, we carefully reviewed the manuscript to eliminate repeated content and improve the overall structure. Redundant descriptions were condensed throughout the Introduction, duplicate explanations of single-item scales were removed, and the **Methods and Analysis** section was comprehensively reorganized according to the Arksey and O'Malley framework. In addition, related methodological information was consolidated into dedicated subsections to avoid repetition while improving the logical flow of the manuscript.

These revisions have substantially reduced redundancy across the abstract, introduction, and methods sections while maintaining the methodological detail appropriate for a scoping review protocol.
