# Supplementary material for: Use of single-item scales in the evaluation of German digital health applications (DiGAs): Scoping review protocol
Source: PLoS One. 2026 Aug 26;21(8):e0356485. doi: 10.1371/journal.pone.0356485 (PMC13514044; doi:10.1371/journal.pone.0356485)
Supplement: Peer review history

# Peer review: Decision Letter 0

**DOI:** 10.1371/journal.pone.0356485.r001

**Authors:** Felix Bongomin

5 Jun 2026

Dear Dr. Wienert,

Thank you for submitting your manuscript to PLOS ONE. After careful consideration, we feel that it has merit but does not fully meet PLOS ONE’s publication criteria as it currently stands. Therefore, we invite you to submit a revised version of the manuscript that addresses the points raised during the review process.

Please submit your revised manuscript by Jul 20 2026 11:59PM. If you will need more time than this to complete your revisions, please reply to this message or contact the journal office at plosone@plos.org. When you're ready to submit your revision, log on to https://www.editorialmanager.com/pone/ and select the 'Submissions Needing Revision' folder to locate your manuscript file.

- A letter that responds to each point raised by the academic editor and reviewer(s). You should upload this letter as a separate file labeled 'Response to Reviewers'.
- A marked-up copy of your manuscript that highlights changes made to the original version. You should upload this as a separate file labeled 'Revised Manuscript with Track Changes'.
- An unmarked version of your revised paper without tracked changes. You should upload this as a separate file labeled 'Manuscript'.

If you would like to make changes to your financial disclosure, please include your updated statement in your cover letter. Guidelines for resubmitting your figure files are available below the reviewer comments at the end of this letter.

If applicable, we recommend that you deposit your laboratory protocols in protocols.io to enhance the reproducibility of your results. Protocols.io assigns your protocol its own identifier (DOI) so that it can be cited independently in the future. For instructions see: https://journals.plos.org/plosone/s/submission-guidelines#loc-laboratory-protocols. Additionally, PLOS ONE offers an option for publishing peer-reviewed Lab Protocol articles, which describe protocols hosted on protocols.io. Read more information on sharing protocols at https://plos.org/protocols?utm_medium=editorial-email&utm_source=authorletters&utm_campaign=protocols.

As the corresponding author, your ORCID iD is verified in the submission system and will appear in the published article. PLOS supports the use of ORCID, and we encourage all coauthors to register for an ORCID iD and use it as well. Please encourage your coauthors to verify their ORCID iD within the submission system before final acceptance, as unverified ORCID iDs will not appear in the published article. *Only*  the individual author can complete the verification step; PLOS staff *cannot*  verify ORCID iDs on behalf of authors.

We look forward to receiving your revised manuscript.

Kind regards,

Felix Bongomin, MB ChB, Ph.D., FECMM

Academic Editor

PLOS One

Journal Requirements:

When submitting your revision, we need you to address these additional requirements.

1. Please ensure that your manuscript meets PLOS ONE's style requirements, including those for file naming. The PLOS ONE style templates can be found at

https://journals.plos.org/plosone/s/file?id=wjVg/PLOSOne_formatting_sample_main_body.pdf and

https://journals.plos.org/plosone/s/file?id=ba62/PLOSOne_formatting_sample_title_authors_affiliations.pdf

2. Thank you for stating the following in the Acknowledgments Section of your manuscript:

“Open Access publication enabled by IU International University of Applied Sciences. The funding was awarded to JW by IU International University of Applied Sciences (https://www.iu.org). No grant number was provided. The sponsor did not play any role in the study design, decision to publish, or preparation of the manuscript.”

We note that you have provided funding information that is not currently declared in your Funding Statement. However, funding information should not appear in the Acknowledgments section or other areas of your manuscript. We will only publish funding information present in the Funding Statement section of the online submission form.

Please remove any funding-related text from the manuscript and let us know how you would like to update your Funding Statement. Currently, your Funding Statement reads as follows:

“Open Access publication enabled by IU International University of Applied Sciences. The funding was awarded to JW by IU International University of Applied Sciences (https://www.iu.org). No grant number was provided. The sponsor did not play any role in the study design, decision to publish, or preparation of the manuscript.”

Please include your amended statements within your cover letter; we will change the online submission form on your behalf.

3. We note that the grant information you provided in the ‘Funding Information’ and ‘Financial Disclosure’ sections do not match.

When you resubmit, please ensure that you provide the correct grant numbers for the awards you received for your study in the ‘Funding Information’ section.

4. Thank you for stating the following financial disclosure:

“Open Access publication enabled by IU International University of Applied Sciences. The funding was awarded to JW by IU International University of Applied Sciences (https://www.iu.org). No grant number was provided. The sponsor did not play any role in the study design, decision to publish, or preparation of the manuscript.”

We note that one or more of the authors is affiliated with the funding organization, indicating the funder may have had some role in the design, data collection, analysis or preparation of your manuscript for publication; in other words, the funder played an indirect role through the participation of the co-authors. If the funding organization did not play a role in the study design, data collection and analysis, decision to publish, or preparation of the manuscript and only provided financial support in the form of authors' salaries and/or research materials, please do the following:

1. Review your statements relating to the author contributions, and ensure you have specifically and accurately indicated the role(s) that these authors had in your study. These amendments should be made in the online form.

2. Confirm in your cover letter that you agree with the following statement, and we will change the online submission form on your behalf:

“The funder provided support in the form of salaries for authors [insert relevant initials], but did not have any additional role in the study design, data collection and analysis, decision to publish, or preparation of the manuscript. The specific roles of these authors are articulated in the ‘author contributions’ section.

5. If the reviewer comments include a recommendation to cite specific previously published works, please review and evaluate these publications to determine whether they are relevant and should be cited. There is no requirement to cite these works unless the editor has indicated otherwise.

[Note: HTML markup is below. Please do not edit.]

Reviewers' comments:

Reviewer's Responses to Questions

**Comments to the Author**

1. Does the manuscript provide a valid rationale for the proposed study, with clearly identified and justified research questions?

Reviewer #1: Yes

**********

2. Is the protocol technically sound and planned in a manner that will lead to a meaningful outcome and allow testing the stated hypotheses?

Reviewer #1: Partly

**********

3. Is the methodology feasible and described in sufficient detail to allow the work to be replicable?

Reviewer #1: No

**********

4. Have the authors described where all data underlying the findings will be made available when the study is complete?

The PLOS Data policy

Reviewer #1: Yes

**********

5. Is the manuscript presented in an intelligible fashion and written in standard English?

Reviewer #1: Yes

**********

Please use the space provided to explain your answers to the questions above and, if applicable, provide comments about issues authors must address before this protocol can be accepted for publication. You may also include additional comments for the author, including concerns about research or publication ethics.

You may also provide optional suggestions and comments to authors that they might find helpful in planning their study.

Reviewer #1: Reviewer Comments

Use of Single-Item Scales in the Evaluation of German Digital Health Applications (DiGAs): Scoping Review Protocol

Thank you for sharing this protocol. The topic is relevant, the literature base is appropriate, and the motivation for the review is clear. My comments below are intended to strengthen the manuscript.

ABSTRACT

1. Introduction sentence needs rewriting: The opening of the abstract is confusing as currently written. Terms like "reimbursable" are used without sufficient context for a general reader and the core message is unclear. Recommend rewriting to better reflect and communicate what is in the introduction.

2. Language implies a systematic rather than scoping review: Bold claims in the abstract suggest a systematic examination of evidence, which overstates the intent of a scoping review. Scoping reviews are exploratory in nature and the language should reflect that.

INTRODUCTION

3. Sentences are too long Several sentences in the introduction are unwieldy and need to be shortened for readability.

4. Single-item scale measurement is not defined: The introduction does not explain or illustrate what a single-item scale actually is. Recommend defining it clearly and directing readers to the mini-narrative review by Lauren Staples: "Single item measures in digital mental healthcare: a mini narrative review of challenges and opportunities."

5. Introduction is biased toward disadvantages: The introduction leans heavily toward the limitations of single-item scales, which mirrors the apparent direction of the review. To present a balanced foundation, dedicate one paragraph to the advantages of single-item scales and a separate paragraph to the challenges.

6. Objectives are buried in prose: The objectives are lost within paragraphs rather than stated clearly and directly. They should be presented in a simple, explicit, standalone format.

7. The scope is too broad — specify the clinical domain: Digital health spans many fields (cardiology, psychiatry, oncology, etc.) and the constructs being measured will differ substantially across them. Mental health differs meaningfully from, say, cardiology, and this distinction matters for both the aims and the search strategy.

METHODS

8. Rewrite using Arksey and O'Malley's six-stage framework as subheadings: The current methods section is repetitive and omits key information. The entire section should be restructured around Arksey's framework, using the following as subheadings:

• Formulation of the research question

• Identifying relevant studies

• Selecting eligible studies (inclusion and exclusion criteria)

• Data charting (extraction and data management)

• Collating, summarising, and reporting results

• Consultation

9. Exclusion criteria are missing: The current version discusses inclusion criteria but omits exclusion criteria entirely. Both must be included under the study selection section.

10. Revise the PCC framework: The Population, Concept, and Context (PCC) framework needs to be revisited and revised. Inclusion and exclusion criteria should each be presented as a subsection with the PCC framework embedded.

11. Critical appraisal section is absent: There is no mention of whether critical appraisal will be conducted. A dedicated section should address this explicitly — whether appraisal will be done, and if not, why not, which is acceptable in a scoping review but must be stated.

12. No section on managing overlapping publications :A section addressing how overlapping or duplicate publications will be identified and handled is missing.

13. Search strategy needs revision: The current strategy searches for DiGAs broadly rather than specifically for single-item scale measurement within DiGA evaluations. This needs to be refocused. Additional concerns:

• The current approach risks producing homogeneous, grey-literature-heavy results

• A librarian should be engaged to access multiple databases systematically

• Be explicit about whether sources are published literature or grey literature

• The search strategy should be included as an appendix

• Sources for screening should be more diverse

14. Explain what the DiGA directory contains: It is not clear what type of information is held in the DiGA directory, how many studies have evaluated DiGAs, or how many of those used single-item scales. This context is needed.

15. Data extraction section is poorly organised: The data extraction section is fragmented and needs to be reorganized clearly, specifying what data will be extracted and how it will be managed.

16. Screening should involve two independent reviewers: Screening and extraction should be performed concurrently by two independent researchers who then compare and reconcile their work. Having one researcher verify the work of another introduces bias and is not standard practice.

17. Specify whether a screening tool will be used: It should be stated whether a tool such as Covidence will be used to manage screening and extraction.

OVERALL / CROSS-CUTTING

18. The central focus of the review is unclear: It is not consistently clear whether the review is about DiGAs themselves or about the use of single-item scales in the evaluation of DiGAs. If it is the latter — which the title suggests — the entire manuscript needs to be adjusted to reflect this focus consistently throughout.

19. Reference 2 is a conference abstract: Fujita-Rohwerder and Sauerland (2022) is a conference abstract, not a full peer-reviewed publication. This should be acknowledged, or a fuller publication substituted if available.

20. Reduce repetition throughout the manuscript: The same content appears in multiple sections. A thorough edit for redundancy across the abstract, introduction, and methods is needed.

**********

what does this mean?). If published, this will include your full peer review and any attached files.

If you choose “no”, your identity will remain anonymous but your review may still be made public.

**Do you want your identity to be public for this peer review?**  For information about this choice, including consent withdrawal, please see our Privacy Policy

Reviewer #1: **Yes:** RAYMOND BERNARD KIHUMURO

**********

[NOTE: If reviewer comments were submitted as an attachment file, they will be attached to this email and accessible via the submission site. Please log into your account, locate the manuscript record, and check for the action link "View Attachments". If this link does not appear, there are no attachment files.]

To ensure your figures meet our technical requirements, please review our figure guidelines: https://journals.plos.org/plosone/s/figures

You may also use PLOS’s free figure tool, NAAS, to help you prepare publication quality figures: https://journals.plos.org/plosone/s/figures#loc-tools-for-figure-preparation.

NAAS will assess whether your figures meet our technical requirements by comparing each figure against our figure specifications.

Attachment Submitted filename: DIGA - comments kbr.docx

# Author comment: Author response to Decision Letter 1

**DOI:** 10.1371/journal.pone.0356485.r002

21 Jul 2026

#####Please also see our submitted file "Response to Reviewer" which contains our formatted responses to each comment. Thank you for your time and effort.#####

Dear academic editor and reviewer,

We sincerely thank you for the time and effort you invested in reviewing our manuscript entitled " Use of single-item scales in the evaluation of German digital health applications (DiGAs): Scoping review protocol" We greatly appreciate the constructive and detailed comments, which have helped us to improve the clarity, methodological transparency, and overall quality of the manuscript.

We have carefully considered every comment and revised the manuscript accordingly. In the point-by-point responses below, each reviewer comment is reproduced in bold, followed by our response and, where appropriate, verbatim excerpts from the revised manuscript to facilitate review. All changes in the revised manuscript have been highlighted using track changes.

We hope that the revisions satisfactorily address all concerns and that the revised manuscript is now suitable for publication. We thank you again for your time and thoughtful feedback and for the opportunity to improve our work.

Sincerely,

the authors

1-Editor Comment:

Please ensure that your manuscript meets PLOS ONE's style requirements, including file naming.

Answer:

We confirm that the revised manuscript has been checked for compliance with the PLOS ONE formatting and style requirements, including manuscript formatting and file naming, in accordance with the journal guidelines.

2 & 3-Editor Comments:

Please remove funding information from the manuscript and provide the revised Funding Statement for the online submission system.

Answer:

We thank the Editorial Office for these clarifications regarding the Funding Statement and Financial Disclosure.

In accordance with the Academic Editor's instructions, we removed all funding-related information from the manuscript. At that stage, we did not modify the online submission system, as the decision letter explicitly instructed us to provide the revised statements in our cover letter and indicated that the journal would update the submission metadata on our behalf.

Following the subsequent editorial request indicating that additional changes to the Funding Information and Financial Disclosure were required, we have now updated the information in the online submission system ourselves to ensure consistency between both sections and to avoid further delays in the review process.

The consolidated Financial Disclosure statement now reads:

Open Access publication was supported by IU International University of Applied Sciences (https://www.iu.org). No research grant was awarded for this study; therefore, no grant number exists. Author JW received Open Access publication support and is employed by IU International University of Applied Sciences. The funder provided support in the form of salaries for author JW, but did not have any additional role in the study design, data collection and analysis, decision to publish, or preparation of the manuscript. The specific role of this author is articulated in the "Contributions" section.

4-Editor Comment:

Clarify the role of the funding organization by confirming that its involvement was limited to financial support (author salary and/or publication costs), revise the Author Contributions accordingly, and provide the requested Financial Disclosure statement.

Answer:

4.1 We thank the Editorial Office for this clarification. We have revised the Author Contributions to provide a more transparent and standardized description of each author's contributions. The revised Author Contributions state:

“JW conceived the study, developed the methodological approach, prepared the original draft of the protocol, and led the manuscript revision, including reviewing and integrating revisions prior to submission. MM contributed to the methodological development of the protocol and provided critical intellectual feedback through review and editing of the manuscript.”

4.2 We thank the Editorial Office for this clarification.

In response to the Academic Editor's previous instructions, we revised the Author Contributions to provide a more transparent and standardized description of each author's role in accordance with the CRediT (Contributor Roles Taxonomy). At that stage, we also confirmed our agreement with the requested Financial Disclosure statement in the cover letter, as the decision letter instructed us not to modify the online submission system and indicated that the journal would update the submission metadata on our behalf.

Following the subsequent editorial request for additional revisions to the Funding Information and Financial Disclosure, we have now updated the online submission system ourselves to ensure that all funding and employment disclosures are presented consistently in a single Financial Disclosure statement.

Accordingly, the revised Financial Disclosure includes the wording:

"The funder provided support in the form of salaries for author JW, but did not have any additional role in the study design, data collection and analysis, decision to publish, or preparation of the manuscript. The specific role of this author is articulated in the "Contributions" section."

The Author Contributions remain unchanged from our previous revision, as they already accurately describe the specific roles of each author.

5-Editor Comment:

If the reviewer comments include a recommendation to cite specific previously published works, please review and evaluate these publications to determine whether they are relevant and should be cited.

Answer:

We carefully evaluated all references suggested by the reviewer. Relevant literature has been incorporated into the revised manuscript where appropriate. References that were not considered directly relevant to the objectives and scope of this protocol were not included.

1-Reviewer Comment:

Introduction sentence needs rewriting: The opening of the abstract is confusing as currently written. Terms like “reimbursable” are used without sufficient context for a general reader and the core message is unclear. Recommend rewriting to better reflect and communicate what is in the introduction.

Answer:

We thank the reviewer for this helpful suggestion. We agree that the opening sentence of the abstract required additional context for readers who may be unfamiliar with the German DiGA framework. We have therefore revised the opening of the introduction to introduce DiGAs more clearly and to explain their reimbursement within the German healthcare system. The revised wording provides the necessary context before introducing the methodological focus of the review.

Revised manuscript (Abstract):

"Digital health applications are increasingly integrated into routine healthcare delivery. In Germany, so-called Digitale Gesundheitsanwendungen (DiGAs) represent a distinct category of patient-facing digital health applications. These applications can be prescribed

by medical doctors and are reimbursed by statutory health insurance schemes once they are listed in the official DiGA directory maintained by the German Federal Institute for Drugs and Medical Devices (BfArM) [1]. The DiGA directory is a regulatory register of all Digital Health Applications that have successfully completed the statutory assessment procedure under §139e SGB V and the German Digital Health Applications Ordinance (DiGAV). It provides structured information on each DiGA, including basic product characteristics (e.g., name, manufacturer, intended medical purpose), functional description, usage conditions, data protection and interoperability features, and information relevant to healthcare providers and patients. Importantly, it also contains references to the scientific evidence submitted for reimbursement approval, including evaluation studies and trial registrations used to demonstrate positive healthcare effects."

2-Reviewer Comment:

Language implies a systematic rather than scoping review: Bold claims in the abstract suggest a systematic examination of evidence, which overstates the intent of a scoping review. Scoping reviews are exploratory in nature and the language should reflect that.

Answer:

We thank the reviewer for this important methodological observation. We agree that the language used throughout the manuscript should accurately reflect the exploratory nature of a scoping review.

In response, we carefully reviewed the manuscript and revised the wording where appropriate to ensure consistency with scoping review methodology. In particular, the review is now consistently presented as a descriptive mapping of the use of single-item scales in DiGA evaluations rather than as a systematic synthesis of evidence. The revised manuscript also explicitly states that quantitative findings will be summarized descriptively, that no meta-analysis is planned, and that no formal critical appraisal of included studies will be undertaken, in accordance with established scoping review methodology.

3-Reviewer Comment:

Sentences are too long Several sentences in the introduction are unwieldy and need to be shortened for readability.

Answer:

We thank the reviewer for this helpful comment. We carefully revised the Introduction to improve readability by shortening several overly long sentences and simplifying the overall structure where appropriate. In addition, we removed redundant wording and reorganized parts of the introduction into shorter, more focused paragraphs. These revisions improve the flow of the text while preserving the intended content and level of detail.

4-Reviewer Comment:

Single-item scale measurement is not defined: The introduction does not explain or illustrate what a single-item scale actually is. Recommend defining it clearly and directing readers to the mini-narrative review by Lauren Staples: "Single item measures in digital mental healthcare: a mini narrative review of challenges and opportunities."

Answer:

We thank the reviewer for this helpful suggestion. We agree that the introduction should clearly define the concept of a single-item scale before discussing its application in DiGA evaluations.

In response, we expanded the Introduction by adding a definition of single-item scales and by providing additional context on their use in digital health interventions. We also incorporated the recommended mini-narrative review by Staples et al. (2026) to contextualize the use of single-item measures in digital mental healthcare.

Revised manuscript (Introduction):

" A single-item scale is a measurement instrument consisting of only one question or statement to assess a particular construct or attribute. Despite their simplicity, these scales can be effective for measuring various aspects, such as symptoms, satisfaction, or perceived health status, particularly in contexts where brevity is essential, such as in digital health intervention [5–7]. For example, a commonly used single-item measure is the global health question: "In general, would you say your health is excellent, very good, good, fair, or poor?" [5]. Recent evidence from digital mental health contexts further supports the practical utility of single-item scales for large-scale screening, outcome monitoring, and integration into digital platforms. This review provides a valuable synthesis of single-item scales use in digital mental healthcare from a clinical effectiveness perspective [8]."

5-Reviewer Comment:

Introduction is biased toward disadvantages: The introduction leans heavily toward the limitations of single-item scales, which mirrors the apparent direction of the review. To present a balanced foundation, dedicate one paragraph to the advantages of single-item scales and a separate paragraph to the challenges.

Answer:

We thank the reviewer for this valuable suggestion. We agree that the introduction should provide a balanced overview of both the advantages and limitations of single-item scales before introducing the rationale for the review.

In response, we revised the Introduction by separating the discussion into two distinct paragraphs. The first paragraph summarizes the practical and methodological advantages of single-item scales, including their suitability for digital health interventions, reduced response burden, and evidence supporting their use for assessing clearly defined constructs. The subsequent paragraph discusses their methodological limitations, including restricted construct coverage, the inability to assess internal consistency, and the importance of careful theoretical justification.

This revised structure provides a more balanced and neutral introduction to the topic and better reflects the exploratory nature of the proposed scoping review.

Revised manuscript (Introduction):

"In this context, single-item scales offer a promising approach to mitigating competing demands between methodological rigor and feasibility. A single-item scale is a measurement instrument consisting of only one question or statement to assess a particular construct or attribute. Despite their simplicity, these scales can be effective for measuring various aspects, such as symptoms, satisfaction, or perceived health status, particularly in contexts where brevity is essential, such as in digital health intervention [5–7]. For example, a commonly used single-item measure is the global health question: "In general, would you say your health is excellent, very good, good, fair, or poor?" [5]. Recent evidence from digital mental health contexts further supports the practical utility of single-item scales for large-scale screening, outcome monitoring, and integration into digital platforms. This review provides a valuable synthesis of single-item scales use in digital mental healthcare from a clinical effectiveness perspective [8]. More recent clinical research further supports the utility of single-item quality-of-life assessments, demonstrating robust associations with well-being, health indicators, and lifestyle-related correlates [6]. Methodological studies further suggest that single-item measures may be appropriate when constructs are unidimensional, concrete, and easily understood by respondents, particularly when minimizing participant burden is a priority [7]."

6-Reviewer Comment:

Objectives are buried in prose: The objectives are lost within paragraphs rather than stated clearly and directly. They should be presented in a simple, explicit, standalone format.

Answer:

We thank the reviewer for this helpful suggestion. We agree that the objectives should be presented in a clear and easily identifiable format.

In response, we restructured the Aims and objectives section by separating the overall aim from the specific objectives and presenting the latter as a standalone list. We also added a separate Expected Outcomes subsection to distinguish the objectives of the review from the anticipated contributions of the study.

Revised manuscript (Aims and objectives – Objectives):

• To identify which DiGA evaluations employ single-item scales.

• To describe the constructs assessed using single-item scales.

• To map the reported rationales, advantages, and limitations associated with their use.

• To identify methodological patterns and gaps in current evaluation practices.

7-Reviewer Comment:

The scope is too broad — specify the clinical domain: Digital health spans many fields (cardiology, psychiatry, oncology, etc.) and the constructs being measured will differ substantially across them. Mental health differs meaningfully from, say, cardiology, and this distinction matters for both the aims and the search strategy.

Answer:

We thank the reviewer for this thoughtful comment. We agree that Digital Health Applications encompass a broad range of clinical indications and that the constructs assessed may vary across therapeutic areas.

However, the objective of this scoping review is methodological rather than clinical. The review does not aim to compare measurement practices between disease areas; instead, it seeks to map the use of single-item scales across DiGA evaluations within the German regulatory framework. Consequently, the use of single-item measures constitutes the primary unit of analysis, whereas

Attachment Submitted filename: Response to Reviewers.docx

# Peer review: Decision Letter 1

**DOI:** 10.1371/journal.pone.0356485.r003

**Authors:** Felix Bongomin

4 Aug 2026

Use of single-item scales in the evaluation of German digital health applications (DiGAs): Scoping review protocol

PONE-D-26-16557R1

Dear Dr. Wienert,

We’re pleased to inform you that your manuscript has been judged scientifically suitable for publication and will be formally accepted for publication once it meets all outstanding technical requirements.

Within one week, you’ll receive an e-mail detailing the required amendments. When these have been addressed, you’ll receive a formal acceptance letter and your manuscript will be scheduled for publication.

An invoice will be generated when your article is formally accepted. Please note, if your institution has a publishing partnership with PLOS and your article meets the relevant criteria, all or part of your publication costs will be covered. Please make sure your user information is up-to-date by logging into Editorial Manager at Editorial Manager® and clicking the ‘Update My Information' link at the top of the page. For questions related to billing, please contact billing support.

If your institution or institutions have a press office, please notify them about your upcoming paper to help maximize its impact. If they’ll be preparing press materials, please inform our press team as soon as possible -- no later than 48 hours after receiving the formal acceptance. Your manuscript will remain under strict press embargo until 2 pm Eastern Time on the date of publication. For more information, please contact onepress@plos.org.

Kind regards,

Felix Bongomin, MB ChB, Ph.D., FECMM

Academic Editor

PLOS One

Additional Editor Comments (optional):

Reviewers' comments:

Reviewer's Responses to Questions

**Comments to the Author**

1. Does the manuscript provide a valid rationale for the proposed study, with clearly identified and justified research questions?

Reviewer #1: Yes

**********

2. Is the protocol technically sound and planned in a manner that will lead to a meaningful outcome and allow testing the stated hypotheses?

Reviewer #1: Yes

**********

3. Is the methodology feasible and described in sufficient detail to allow the work to be replicable?

Reviewer #1: Yes

**********

4. Have the authors described where all data underlying the findings will be made available when the study is complete?

The PLOS Data policy

Reviewer #1: Yes

**********

5. Is the manuscript presented in an intelligible fashion and written in standard English?

Reviewer #1: Yes

**********

Please use the space provided to explain your answers to the questions above and, if applicable, provide comments about issues authors must address before this protocol can be accepted for publication. You may also include additional comments for the author, including concerns about research or publication ethics.

You may also provide optional suggestions and comments to authors that they might find helpful in planning their study.

Reviewer #1: The Authors responded to the reviewer's comments to the reviewer's satisfaction. The Protocol now reads well

**********

what does this mean?). If published, this will include your full peer review and any attached files.

If you choose “no”, your identity will remain anonymous but your review may still be made public.

**Do you want your identity to be public for this peer review?**  For information about this choice, including consent withdrawal, please see our Privacy Policy

Reviewer #1: **Yes:** RAYMOND BERNARD KIHUMURO

**********

# Editor report: Acceptance letter

**DOI:** 10.1371/journal.pone.0356485.r004

**Authors:** Felix Bongomin

PONE-D-26-16557R1

PLOS One

Dear Dr. Wienert,

I'm pleased to inform you that your manuscript has been deemed suitable for publication in PLOS One. Congratulations! Your manuscript is now being handed over to our production team.

At this stage, our production department will prepare your paper for publication. This includes ensuring the following:

* All references, tables, and figures are properly cited

* All relevant supporting information is included in the manuscript submission,

* There are no issues that prevent the paper from being properly typeset

You will receive further instructions from the production team, including instructions on how to review your proof when it is ready. Please keep in mind that we are working through a large volume of accepted articles, so please give us a few days to review your paper and let you know the next and final steps.

Lastly, if your institution or institutions have a press office, please let them know about your upcoming paper now to help maximize its impact. If they'll be preparing press materials, please inform our press team within the next 48 hours. Your manuscript will remain under strict press embargo until 2 pm Eastern Time on the date of publication. For more information, please contact onepress@plos.org.

You will receive an invoice from PLOS for your publication fee after your manuscript has reached the completed accept phase. If you receive an email requesting payment before acceptance or for any other service, this may be a phishing scheme. Learn how to identify phishing emails and protect your accounts at https://explore.plos.org/phishing.

If we can help with anything else, please email us at customercare@plos.org.

Thank you for submitting your work to PLOS One and supporting open access.

Kind regards,

PLOS ONE Editorial Office Staff

on behalf of

Dr. Felix Bongomin

Academic Editor

PLOS One
